# Functional Films from Silica/Polymer Nanoparticles

**DOI:** 10.3390/ma7053881

**Published:** 2014-05-15

**Authors:** Tânia Ribeiro, Carlos Baleizão, José Paulo S. Farinha

**Affiliations:** CQFM—Centro de Química-Física Molecular and IN—Institute of Nanoscience and Nanotechnology, Instituto Superior Técnico, Universidade de Lisboa, 1049-001 Lisboa, Portugal; E-Mails: tania.ribeiro@tecnico.ulisboa.pt (T.R.); carlos.baleizao@tecnico.ulisboa.pt (C.B.)

**Keywords:** silica-polymer nanoparticles, functional coatings, hybrid films, coupling agents, film formation

## Abstract

High performance functional coatings, based on hybrid organic/inorganic materials, are being developed to combine the polymer flexibility and ease of processing with the mechanical properties and versatility of inorganic materials. By incorporating silica nanoparticles (SiNPs) in the polymeric matrices, it is possible to obtain hybrid polymer films with increased tensile strength and impact resistance, without decreasing the flexural properties of the polymer matrix. The SiNPs can further be used as carriers to impart other functionalities (optical, *etc.*) to the hybrid films. By using polymer-coated SiNPs, it is possible to reduce particle aggregation in the films and, thus, achieve more homogeneous distributions of the inorganic components and, therefore, better properties. On the other hand, by coating polymer particles with silica, one can create hierarchically structured materials, for example to obtain superhydrophobic coatings. In this review, we will cover the latest developments in films prepared from hybrid polymer/silica functional systems.

## 1. Introduction

Hybrid organic/inorganic materials combine the rigidity and high thermal stability of the inorganic material with the flexibility, ductility and processability of the organic polymers. In recent years, silica has been increasingly used in this type of material. Silica-polymer hybrid materials have received much attention, due to their wide application in adhesion, biomaterials, protective coatings, composites, microelectronics, thin-films, *etc*. [1]. Hybrid materials for coating applications can exhibit properties, such as optical transparency, specific electrical and mechanical behavior, thermal and weathering resistance, abrasion and impact resistance [1]. Silica nanoparticles (SiNPs) have many interesting properties, including high mechanical strength, permeability, thermal and chemical stability, a relatively low refractive index and a high surface area. The incorporation of SiNPs in polymer films is also known to enhance the film mechanical properties and to reduce its thermal degradation at high temperature, also improving its insulation properties, and increasing the barrier properties to solvents and volatile products [2].

SiNPs are used in hybrid coatings mainly in two forms: colloidal and fumed silica. Fumed silica, produced via flame hydrolysis, has been frequently used due to the significantly lower cost compared to colloidal silica. However, when dispersed, fumed silica has a tendency to irreversibly aggregate. The preparation of colloidal SiNPs usually follows the method introduced by Stöber [3] that consists of the hydrolysis and condensation of a silica precursor, such as tetraethyl orthosilicate (TEOS), in basic conditions. Using this method, the particle size distribution is relatively narrow and tunable to different diameters, ranging from 20 nm to 1 μM. The silica content of the films must be optimized in order to simultaneously increase the tensile strength and impact resistance, without decreasing its flexural properties. A silica content of *ca.* 10 wt% is usually found to give the best film properties [4,5].

Silica-polymer hybrid materials can be obtained by simply mixing organic and inorganic components; however, it is usually difficult to obtain a homogeneous mixture of silica and organic polymer, due to the formation of SiNP aggregates that strongly affect the properties of the hybrid material. This is in part due to the formation of hydrogen bonds between SiNPs through the silanol groups present on their surface [6]. One way to enhance the compatibility between the polymer and the SiNPs is by functionalizing the silica surface using coupling agents that not only improve the compatibility between organic and inorganic phases, but also enhance the interaction between the components at the interface level. A different approach to functionalize SiNPs uses the high energies of plasma lasers to improve the compatibilization with polymer matrices. The energetic ions from the plasma laser break the Si–C and Si–O bonds in the silane surface groups, creating active sites that can react with the surrounding polymer matrix [7,8].

This review will emphasize the preparation of high performance functional coatings, based on hybrid organic/inorganic materials. The first part of the review will focus the importance of using coupling agents to enhance the compatibility between the organic and inorganic parts of the hybrid material. In the second part, different methods to prepare hybrid silica-polymer materials will be described. Finally, we will present recent reports of functional hybrid coatings.

## 2. Coupling Agents

The use of coupling agents is essential to obtain homogeneous mixtures between the organic and inorganic phases, by enhancing the interaction between the components at the interface level. Table 1 shows a number of typical silane coupling agents of the type [R^1^-Si(OR^2^)_3_], where R^1^ is a functional group, such as aminopropyl or mercaptopropyl, and R^2^ is a methyl or ethyl group, which are commonly used in the preparation of hybrid silica-polymer materials for high performance coatings. In particular, 3-methacryloxypropyl trimethoxysilane (MPS), 3-methacryloxypropyl triethoxysilane (MPTES), 3-aminopropyl triethoxysilane (APTES) and *N*-[3-(trimethoxysilyl) propyl] aniline (PATMS) are examples of molecules that are used to form siloxane bonds via the hydrolysis of the alkoxy groups. MPS has been widely used to improve the compatibility between silica and acrylic monomers, providing polymerization grafting sites for monomers, such as butyl methacrylate (BMA) [9], methyl methacrylate (MMA) [10,11] or acrylic acid (AA) [12], taking advantage of the acryloyl group. The trialkoxy silane groups of MPS allow the covalent bonding to the surface of SiNP or to silica precursors that can further hydrolyze and condensate to form SiNPs (sol-gel reaction). For example, a recent work by Morales-Acosta *et al.* [10] showed the preparation of poly(methyl methacrylate)-MPS-silica (PMMA-MPS-SiNP) thin films by a modified sol-gel technique, in which MMA was added to a previous prepared solution of TEOS-MPS. The authors showed the contribution of MPS to the formation of homogeneous PMMA-MPS-SiNP hybrid films with no organic–inorganic phase separation, optical transparency and a tunable refractive index.

**Figure 1.**
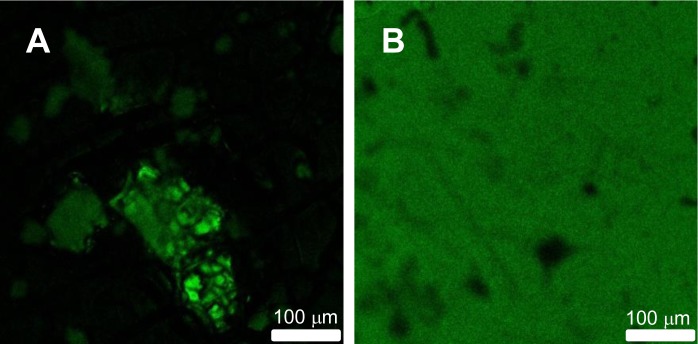
Laser scanning confocal microscope images (0.5 × 0.5 mm) of films obtained by blending water dispersions of fluorescent SiNP with poly(butyl methacrylate) (PBMA) nanoparticles of a 100-nm diameter using the same silica volume fraction as in the core-shell particles show aggregates of fluorescent silica particles and large dark polymer domains (**A**). Films cast from core-shell water dispersions show a homogeneous fluorescence distribution (**B**). Both films were cast from a water dispersion, dried at 32 °C, and annealed at 90 °C for 1 h. Reprinted with permission from [9]. Copyright 2009, American Chemical Society.

Our group used a different approach to enhance the compatibility between the organic and inorganic parts of the film [9]. We prepared core-shell hybrid NPs with a silica core and a poly(butyl methacrylate) (PBMA) shell, where the silica core has a fluorescent dye (a perylenediimide derivative) incorporated in the network, which was used to probe the homogeneity of the final film. In this case, MPS was covalently immobilized on the surface of the SiNPs and then emulsion polymerization was used to grow a PBMA shell. Films obtained from these hybrid core-shell particles have better properties than the films obtained by mixing PBMA nanoparticles with the same content of pure SiNPs. The reason for this is that in the first case, the silica is homogeneously dispersed in the polymer matrix formed from the particle shells, while in the polymer/silica blend films, the silica particles tend to aggregate and segregate from the material. This was observed by laser scanning confocal fluorescence microscopy (LSCFM) in films cast from water dispersions and annealed above their glass transition temperature (*T*_g_) (Figure 1). Films obtained in the same casting conditions by blending water dispersions of fluorescent SiNPs and PBMA nanoparticles of a 100-nm diameter (with the same silica volume fraction as for the core-shell particles) showed that the silica nanoparticles are segregated from large polymer domains (Figure 1A), while films cast from core-shell water dispersions showed a homogeneous distribution of the fluorescent silica cores, except for some drying defects (Figure 1B).

## 3. Hybrid Silica-Polymer Materials for High Performance Coatings

There are three general approaches to obtain silica-polymer hybrid materials: (i) blending the two components by mixing the silica structure (usually, SiNPs) and the polymer; (ii) using a sol-gel process to produce silica *in situ* in the presence of a preformed organic polymer or simultaneously during the polymerization; and (iii) polymerizing the organic monomers in the presence of pre-formed silica structures (e.g., SiNP) [1]. The organic part can thus be introduced as a precursor (monomer or oligomer), a linear polymer or a polymer network, while the inorganic part could be introduced as a precursor or preformed structure. In Table 2, some examples of the three types of approaches and the corresponding organic composition are shown. All of these examples include hybrid materials applied to high performance coatings.

Although the blending approach is the faster and simpler method to prepare hybrid materials, it is difficult to obtain hybrid films with good mechanical properties and high optical transparency, due to the formation of silica aggregates. To overcome this problem, Chau *et al.* [17] prepared silica-PMMA hybrid coatings with SiNPs modified at the surface with MPS to enhance the adhesion between the SiNPs and PMMA matrix, obtaining transparent hybrid films with good thermal stability and hardness. Furthermore, using the blending process, Magalhães *et al.* [23] prepared self-cleaning hybrid coatings using functionalized fumed silica. The use of fumed silica with an acrylate functionalization at the surface facilitated the compatibility and promoted interaction with the polymer matrix during the blending process.

The preparation of SiNPs by the sol-gel method involves the hydrolysis and condensation of a silica precursor, most frequently TEOS, and the formation of a silica network. The sol-gel approach is commonly used to introduce silica onto the surface of polymer particles, for example, as a silica shell [30], or by coating the polymer with SiNPs [32]. This method has a number of disadvantages that limit the use of silica coated particles in practical applications. First, the sol-gel process uses ethanol or another alcohol to slow down and control the hydrolysis and condensation reactions of silica precursor, compromising the stability of the aqueous polymer dispersions. As an alternative, the polymer particles can be stabilized in ethanol prior to the sol-gel process, but this involves a complex and expensive industrial process. The second drawback is the presence of surfactants resulting from the emulsion polymerization process used to prepare the polymer particle dispersion, which tend to adsorb onto the polymer particle and hinder the grafting of silica onto its surface.

**Figure 2.**
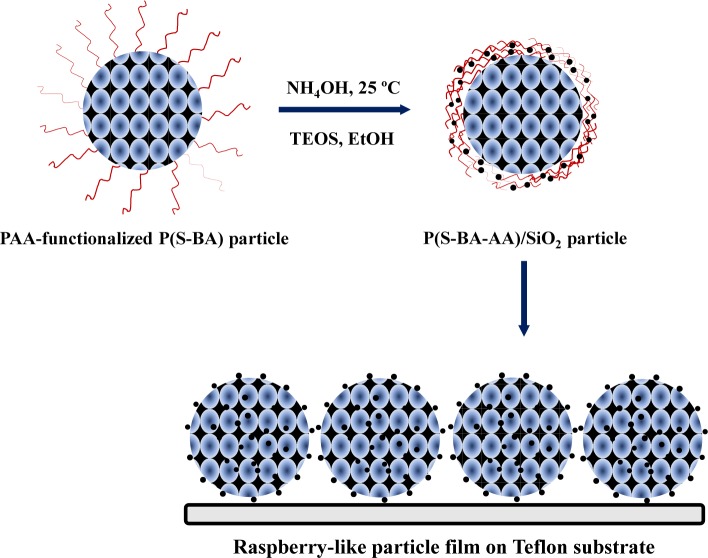
Schematic illustration of film formation from P(S-BA-AA)/SiNP raspberry-type particles obtained by sol-gel coating of polymer particles with TEOS, tetraethyl orthosilicate [32].

In order to overcome these disadvantages, two different approaches can be implemented. One possibility is to use emulsifier-free emulsion polymerization to prepare the polymer, as for example in the work reported by Liu *et al.* [32], where hybrid films were prepared with raspberry-like particles composed of poly(acrylic acid)-functionalized poly(styrene-butyl acrylate) particles covered with SiNPs (Figure 2). The SiNPs were anchored on the surface of the polymer particles by a sol-gel reaction, using TEOS and ethanol. The authors obtained hybrid films that had better mechanical and fire-retardant properties, as well as water resistance than films of the pure polymer.

**Figure 3.**
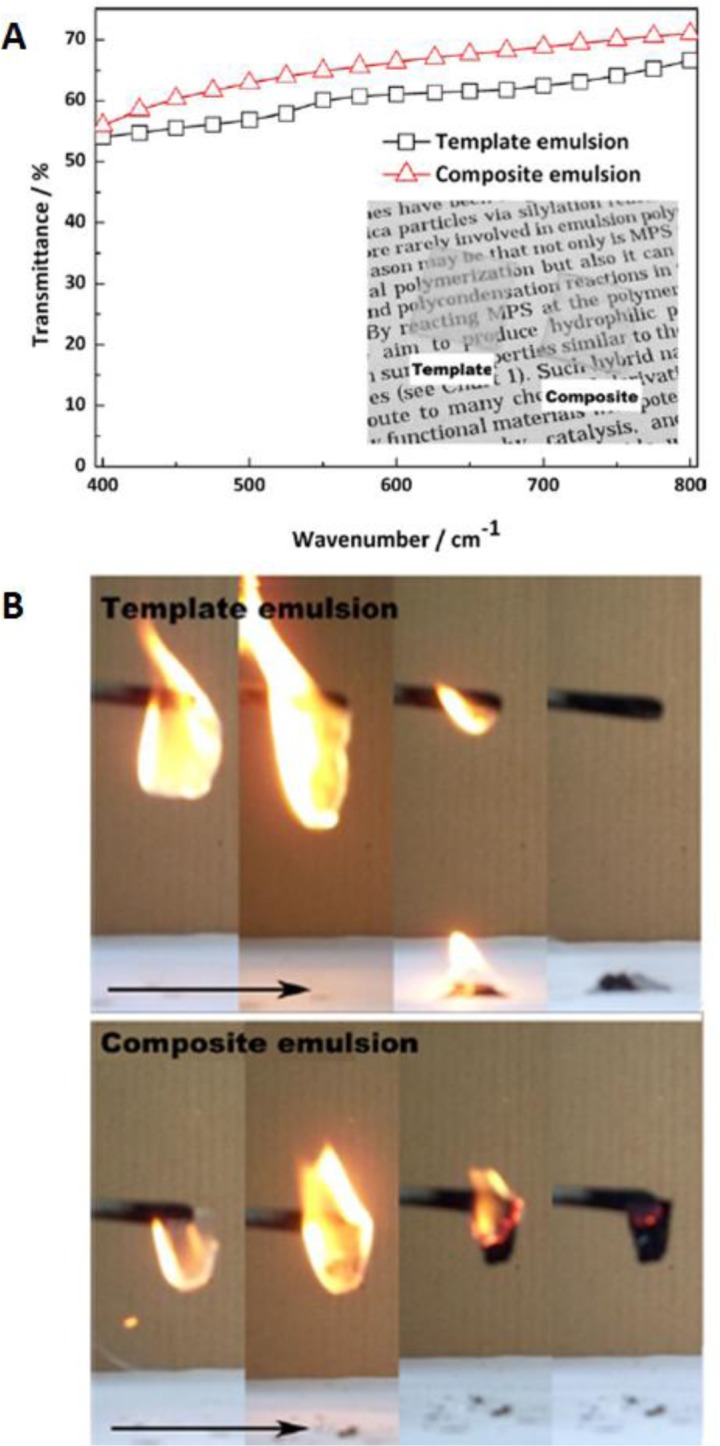
(**A**) The transmittance spectra of the films made from template emulsion and composite emulsion; (**B**) the burning behavior of the template emulsion film and composite emulsion film. Reprinted with permission from [30]. Copyright 2013, Wiley Periodicals, Inc.

A different strategy was recently reported by Xia *et al.* [30], who prepared poly(styrene)-silica core-shell particles by synthesizing the polymer core by emulsion polymerization and then coating with silica, via a sol-gel reaction with no addition of ethanol and without removing the surfactant. Films obtained from these particles were found to have better optical and flame-retardant properties than pure polymer films (Figure 3).

Generally, there are two main approaches to chemically attach polymer chains onto the surface of the SiNPs. In the “grafting-from” method, the monomer is mixed directly with the SiNPs and polymerized from the surface. The second method is the “grafting-to” approach, where the polymer chains, carrying reactive groups at the end or side chains, are covalently coupled to the surface of the particles. Both methods can be used to modify the particle surface with easily controlled polymer chain density. The encapsulation of SiNPs with grafted chains avoids the desorption of the polymer and provides a much better long-term stability of the hybrid structures [1].

The “grafting-from” method has been the most used in the synthesis of hybrid materials for functional coatings, because it is more versatile in terms of the type and size of the polymers, it is easier to implement and usually yields better coverage densities than the “grafting-to” method. The structures prepared through polymerization use pre-formed SiNPs from which the polymer chain is grown by polymerizing monomer. The encapsulation of SiNPs with a polymer shell has been reported using different types of polymerization, such as dispersion [34], emulsion [35,36], miniemulsion [37,38] and atom transfer radical [39,40] polymerization.

Other less common methods include radiation miniemulsion polymerization, used by Ge *et al.* [16] to prepare raspberry SiNP/polystyrene core-shell particles, for superhydrophobic adhesive films. Here, the miniemulsion containing submicron silica particles was irradiated with ^60^Co γ-rays to initiate the polymerization of styrene, yielding hybrid particles with hydrophobic poly(styrene) (PS) latex nanoparticles on the surface of submicron silica particles.

**Figure 4.**
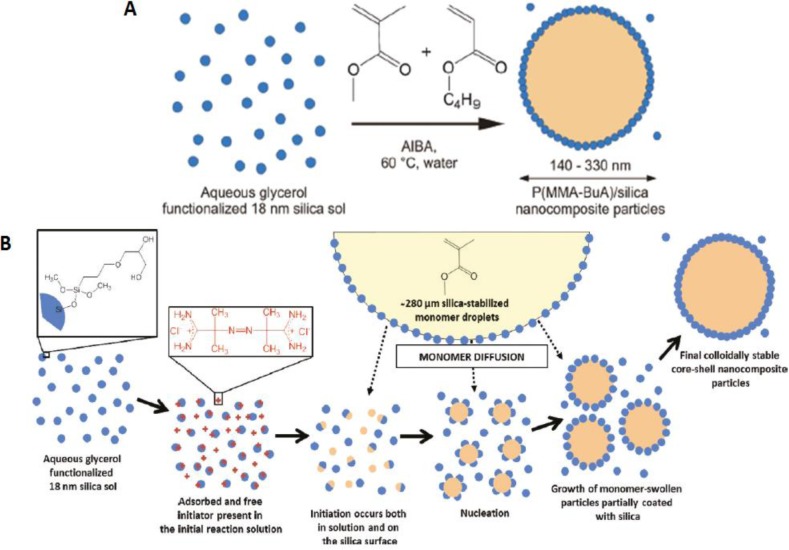
(**A**) Schematic representation of the surfactant-free synthesis of colloidal nanocomposite particles by aqueous emulsion copolymerization; (**B**) schematic representation of the mechanism of formation of colloidal, stable poly(acrylate)/silica particles. Blue circles indicate silica particles; orange regions show polymer, and the 2,2’-azobis(2-methylpropionamide) dihydrochloride (AIBA) initiator is represented by red “+”. Reprinted with permission from [26]. Copyright 2011, American Chemical Society.

Pickering emulsion polymerization is another method that has been used to prepare organic/inorganic nanocomposites, with a polymer core and an inorganic shell. In this process, the emulsion is stabilized by inorganic nanoparticles, thus avoiding the use of conventional low-molecular-weight surfactants [41–44]. For example, Armes *et al.* [26,28] prepared polymer-silica core-shell raspberry structures of poly(styrene-*co*-*n*-butyl acrylate)/silica and poly(methyl methacrylate-*co-n*-butyl acrylate)/silica from a glycerol-functionalized silica sol [26,28]. The mechanism proposed by the authors involves the initiation of the polymerization, both in solution and on the silica surface (Figure 4A,B), leading to the nucleation of polymer particles, which grow partially coated with the SiNP to form stable core-shell particles. The dispersions yielded coatings with good transparency, scratch and mechanical resistance.

**Figure 5.**
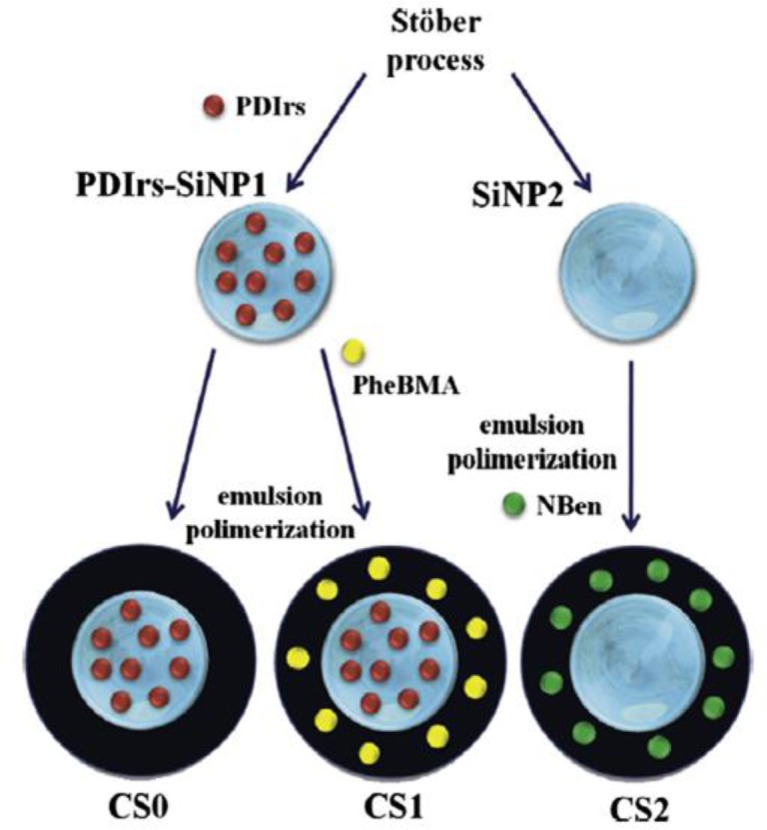
Pure-silica nanoparticles (SiNP2) and silica nanoparticles doped with a perylene derivative (PDIrs) were used as seeds to obtain core-shell hybrid nanoparticles with a silica core and a poly(butyl methacrylate) (PBMA) shell (CS0). The polymer shell of the hybrid particles, CS1 and CS2, was labeled with a phenanthrene derivative, PheBMA, and N-benzophenone (NBen), respectively. Reprinted with permission from [13]. Copyright 2013, Elsevier, Inc.

Our group has reported the preparation of silica-poly(butyl methacrylate) core-shell particles, using seeded semi-continuous emulsion polymerization. Here, the monomer and the initiator are added continuously to an emulsion containing the SiNP surface modified with MPS, as previously described [9,13]. A thick polymer shell could be obtained, with the films cast directly from the particles dispersed in water showing good flexibility and mechanical properties. We also demonstrated the possibility to label the particle silica core with one fluorescent dye (a perylenediimide derivative) and the polymer shell with another (either a phenanthrene derivative, PheBMA, or an N-benzophenone derivative (NBen)) (Figure 5).

## 4. Film Formation

The need to replace organic-solvent-based coatings by more environmentally friendly systems has triggered the development of different strategies to obtain high performance waterborne coatings [1,45–47]. Waterborne coatings use water as a solvent, which makes them more eco-friendly and easy to apply. They are currently available for many different applications, including wood coatings, furniture coatings, plastic coatings, printing inks, *etc*. [1]. The incorporation of silica into waterborne coatings enhances their scratch and abrasion resistance and increases the number of possible applications. The organic part of hybrid waterborne coatings is commonly composed by acrylate [21,25,26,29] or urethane resins [5,48].

**Figure 6.**
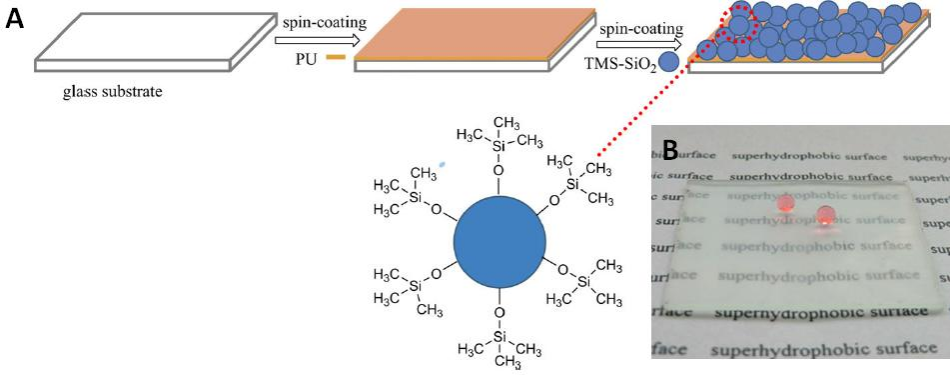
(**A**) The preparation process of the polyurethane/trimethylsiloxane functionalized silica nanoparticle (PU/TMS-SiNP) coating; (**B**) image of the PU/TMS-SiNP superhydrophobic surface on glass substrate (spin-coated with 3 wt% TMS-SiNP). Reprinted with permission from [52]. Copyright 2013, Wiley Periodicals, Inc.

The most common way to prepare hybrid films involves two steps. The first step consists of casting, spreading, spin-coating or dip-coating [11,31,49] the dispersion onto a substrate and, the second step, of drying the wet film, evaporating the solvent at room temperature or in an oven at a specific temperature [12,13,17,21,26–28,30,32,50,51]. In an approach using spin-coating, Wang *et al.* [52] recently presented the preparation of a super-hydrophobic surface by spreading trimethylsiloxane functionalized SiNP (TMS-SiNP) dispersions onto a polyurethane (PU) layer (Figure 6A). The obtained super-hydrophobic coatings were stable and almost transparent (Figure 6B). A similar approach was used in the fabrication of a super-hydrophobic and antireflective coating [53] using layer-by-layer deposition of polyelectrolytes on glass substrates, followed by the spin coating of a superhydrophobic sol-gel that was prepared by hydrolyzing TEOS and then reacting it with hexamethyldisilazane. The main disadvantage of both of these approaches is the non-homogeneous distribution of the inorganic component in the film, contrary to films prepared from hybrid nanoparticles [14,47].

**Figure 7.**
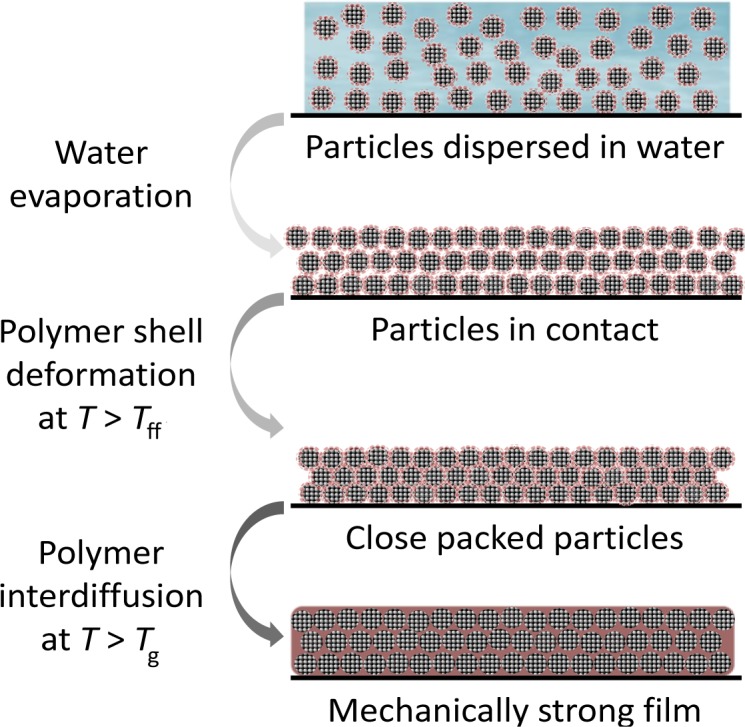
Schematic representation of the steps in the preparation of a hybrid film. A water dispersion of the particles is cast onto a substrate and dried. When the particles come into contact, the polymer shell (in red) deforms (at a temperature above the minimum film formation temperature, *T*_ff_) to occupy the voids between particles, yielding a close-packed arrangement of the particles. If the film is annealed above the glass transition temperature of the polymer shell, *T*_g_, the polymer shell diffuses across the particle-particle boundary to form a mechanically strong film.

The more common procedure to prepare waterborne hybrid films starts with casting the water dispersion of the particles onto a substrate and drying at a temperature above the minimum film formation temperature, *T*_ff_, which is close to the glass transition temperature of the polymer in the presence of water. At this point, the particles form a densely close-packed arrangement, and the polymer shell deforms through a combination of capillary, osmotic and surface forces that overcome the elastic modulus of the polymer, occupying the voids between particles to form a film. A mechanically strong film is then obtained by interdiffusion of the polymer chains in the particle shell across the interfaces between adjacent particles, a process driven by the increase in entropy associated with the healing of these interfaces (Figure 7).

The last step in the mechanism of film formation in hybrid silica-polymer coatings can be studied using the techniques developed for studying polymer interdiffusion across the interfaces in films cast from polymer colloidal dispersions (latex). Among this, Förster resonance energy transfer (FRET) has been widely used [46,54] to study the effects of different factors on latex film formation, such as temperature [55], composition [56], moisture [57], coalescing aids [58–61], polar groups at the latex surface [62,63] and the presence of filler particles [21,27,64]. The use of this technique requires that the polymer is labeled with a small amount (typically less than 1 mol%) of two different dyes: a fluorescent energy donor dye and an energy acceptor dye, for which the fluorescence spectra of the donor overlaps the absorption spectra of the acceptor [65]. The films are then prepared from a blend of donor-labeled particles and acceptor-labeled particles. As the donor and acceptor-labeled polymer chain mix due to diffusion across the inter-particle boundaries, FRET occurring from the donor to the acceptor can be detected by measuring the fluorescence decay of the donor [66], allowing the determination of the evolution of interdiffusion in the film.

**Figure 8.**
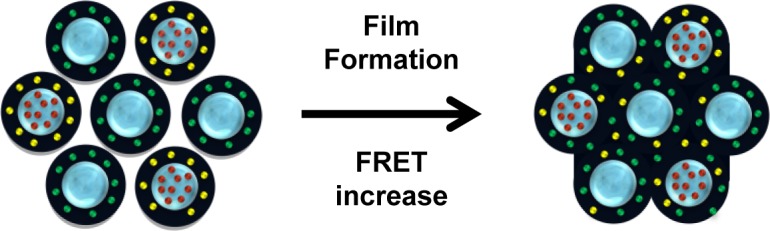
Cartoon showing film formation with a 1:1 weight ratio of core-shell particles with PheBMA (yellow dots) in the polymer shell and a perylene derivative (red dots) in the silica core; and core-shell particles with NBen (green dots) in the polymer shell. Reprinted with permission from [13]. Copyright 2013, Elsevier, Inc.

For example, our group labeled the polymer shell of hybrid nanoparticles with a silica core with either 9-phenanthryl butyl methacrylate (PheBMA) [67] or 4′-dimethylamino-2-acryloxy 5-methyl benzophenone (NBen) [68], following the diffusion of the shell polymer chains by FRET [13,55] (Figures 7 and 8). By evaluating FRET from dynamic fluorescence measurements in film cast from mixed dispersions of donor and acceptor labeled particles [66], it is possible to measure the polymer interdiffusion and understand the dynamic of film formation for silica-polymer nanoparticles [13]. We showed that during film formation the interdiffusion of polymer grafted to a silica particle is not significantly decreased by the silica core, thus explaining the excellent properties of the photoactive high performance coatings formed with this type of particle.

A final curing step can also occur, using radiation [15,19,48,69] (either UV or electron beam (EB)), to crosslink or polymerize hybrid structures on a substrate. The UV curing process involves a photochemical reaction in which the hybrid structures contain a small amount of photoinitiator that, when exposed to UV light, induce the crosslinking of the film. The EB curing process occurs by exposing the film to low-energy electrons that induce the crosslinking of the film. A very recent work of Kumar *et al.* [69] shows the application of this method on the fabrication of hybrid films using an epoxy acrylate resin and surface-modified silica nanoparticles. Zhang *et al.* [48] synthesized waterborne UV-curable polyurethane/silica hybrids using an aqueous silica sol. More recently, in order to enhance the homogeneity and compatibility between the silica nanoparticles and the polyurethane dispersion, the same group used colloidal silica surface-functionalized with poly(ethylene glycol) monomethyl ether methacrylate (PEGMA) to obtain polyurethane/silica hybrids [5]. The PEGMA containing the methacrylate group on the silica surface can be involved in the photopolymerization with the methacrylate group of the polyurethane termini, which becomes grafted to the silica particle surface.

## 5. Coatings Prepared from Hybrid Polymer/Silica Nanoparticles

Hybrid coatings of polymer and SiNPs not only combine the flexibility and easy processing of polymers with the hardness of SiNPs, but can also have a variety of other properties by using the versatility of SiNP to carry catalysts, dyes, drugs, *etc.* Such films can be used in different coating applications with properties, such as being hydrophobic [16,31,49,50,53,70], amphiphobic [51,71], anticorrosion [22], conductive [72], anti-refection [73,74], photoactive [9,13], *etc.*

One example of the use of SiNPs in functional high performance coatings is the preparation of superhydrophobic coatings [50,53], for example using raspberry nanoparticles [16,31,49,75] to obtain a multi-size surface roughness that mimics the topology of self-cleaning plant leaves, with a high water contact angle. Ming *et al.* [75] reported the fabrication of hydrophobic films from silica-based raspberry-like particles prepared from small SiNPs covalently grafted onto larger silica particles on an epoxy-based polymer matrix. Due to the hydrophilic nature of SiNPs, a layer of monoepoxy-end-capped poly(dimethylsiloxane) (PDMS) was grafted onto the raspberry-like particles to give the film surface superhydrophobic properties. Recent works report the fabrication of hybrid raspberry particles, including SiNP-coated polymer particles (with the SiNPs surface modified with hydrophobic dodecyltrichlorosilane [49] or PDMS [31]) and polymer nanoparticle-coated silica particles (in this case, if a hydrophobic polymer is used, such as PS, there is no need for hydrophobization) [16].

**Figure 9.**
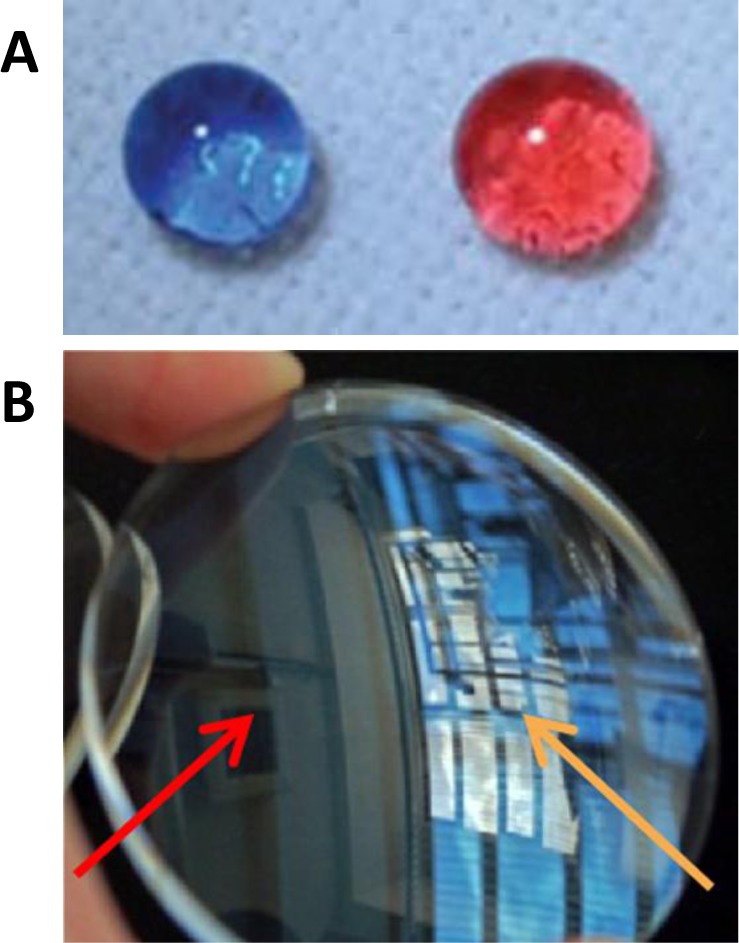
Photographs of examples of (**A**) amphiphobic (the droplets represent blue-colored water and red-colored hexadecane). (reprinted with permission from [71]; copyright 2013, Wiley-VCH Verlag GmbH & Co.); (**B**) anti-reflection coatings formed using hybrid silica-polymer structures (reprinted with permission from [73]; copyright 2013, Elsevier, Inc.).

Another promising application of hybrid nanoparticle films is in the preparation of amphiphobic (or omniphobic) coatings, which have the ability to repel both water and oil (showing a contact angle greater than 150° to both). These can be useful for self-cleaning and anti-sticking corrosion-resistance surfaces. These coatings are, however, difficult to prepare on flat substrates, such as glass windows or metal sheets [51], and hybrid nanoparticles can provide the necessary topological features. There are just a few reports of super-amphiphobic coatings prepared with hybrid nanoparticles [51,71]. Lin *et al.* [71] prepared an interesting super-amphiphobic surface with a self-healing ability to auto-repair from chemical damage, from a coating of poly(vinylidene fluoride-*co*-hexafluoropropylene) (PVDF-HFP), fluoroalkyl silane (FAS) and FAS-modified SiNPs that showed stability to strong acids and bases, ozone, *etc*. (Figure 9A). Xiong *et al.* [51] prepared hybrid particles with a silica core and a shell of poly(2-perfluorooctylethyl methacrylate) and poly(acrylic acid) coronal chains, which were cast onto partially-cured epoxy glues to produce rough particulate coatings, also with amphiphobic properties.

Hybrid coatings for use in the petrochemical industry, for example, must not only combine the flexibility of the polymer component with the hardness and increased chemical resistance imparted by SiNPs, but also avoid the accumulation of static electricity in oil tanks, preventing corrosion and the generation of static charges. In a recent report [22], coatings prepared by blending polystyrene with hybrid core-shell particles composed of a silica core doped with dodecylbenzenesulfonic acid (DBSA) and a conducting polyaniline (PANI) shell, showed antistatic and anticorrosion properties, resulting from the synergistic effects of gas barrier properties and redox catalytic capabilities.

Other applications of conducting polymer coatings are in the fields of organic solar cells, flexible electronics or electrochromic devices. Here, silica can also enhance the mechanical strength and adhesion of the films, as reported by Mandler *et al.* [72], who developed a one-step electrodeposition approach, where polypyrrole (ppy) and silica were deposited from an acidic solution consisting of pyrrole and tetramethoxysilane (TMOS).

Coatings containing hybrid nanoparticles can be used to modify the optical properties of polymer surfaces. For example, polymers, such as poly(methyl methacrylate) (PMMA), polyethylene terephthalate (PET) and polycarbonate (PC), are widely used as an alternative to glass in many applications, such as lenses, displays or photoactive devices, due to their optical properties, mechanical flexibility, light weight, impact resistance and low cost. These materials are commonly coated to improve their impact and abrasion resistance and to minimize reflections from the substrate [76]. The use of SiNPs can protect the substrate against mechanical abrasion and, by tuning their porosity, control the refractive index to achieve antireflection (AR) properties (e.g., using mesoporous SiNPs the refractive index value can decrease) [73]. For example, a single layer of mesoporous SiNPs (Figure 9B) was deposited in a silicate binder solution (with TEOS, isopropanol and hydrochloric acid) to obtain an AR coating for polycarbonate lens that reduced the reflectance to less than 1% [73]. Yun *et al.* [74] also reported an AR nanoarray structure of silica nanoparticles, cast on highly flexible PET substrates by a vacuum coating method, where the reflection and transmission of the AR layer could be controlled by the dimension and average distance of the SiNP on the surface.

**Figure 10.**
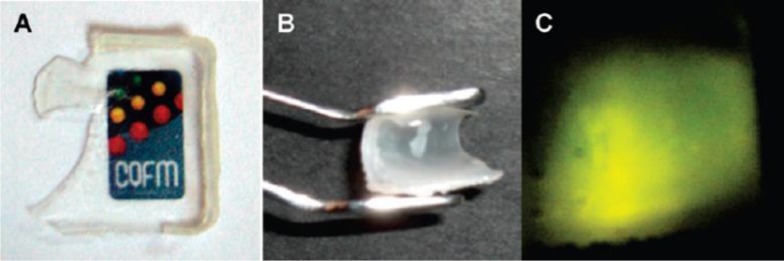
Images of a core-shell film obtained after 60 min of annealing at 90 °C, showing (**A**) transparency; (**B**) flexibility; and (**C**) strong fluorescence emission under excitation at λ_exc_ = 450 nm. Reprinted with permission from [9]. Copyright 2009, American Chemical Society.

The use of SiNPs as hosts for dyes in photoactive hybrid coatings and paints has several advantages compared to the use of dyes directly in the polymer. There is less dye aggregation, better temperature stability and increased dye photostability, due to an oxygen shielding effect. Our group was the first to prepare SiNPs doped with perylenediimide derivatives, by the Stöber method, with a wide range of diameters [9]. The dyes were covalently incorporated in the silica network during the synthesis, with the silica particles being surface modified and covered with a polymer shell by emulsion polymerization. The dispersions of core-shell nanoparticles with a silica fluorescent core and a methacrylate shell [9,13] were used to cast hybrid photoactive high performance coatings. The films showed good transparency, flexibility and strong fluorescence emission under appropriate excitation (Figure 10).

## 6. Conclusions and Outlook

Coatings based on hybrid polymer/silica materials combine the flexural properties and processability of the polymer with a varied array of properties that can be imparted by the inorganic components. Silica nanoparticles (SiNPs) are among the most used for hybrid coating applications. These not only provide increased tensile strength, scratch resistance and impact resistance, but are also an extremely versatile vehicle to incorporate other components, which, in turn, open the possibility to obtain coatings with a wide range of new properties. Although examples of such functional coatings (e.g., anticorrosion, conductive, anti-refection, photoactive, *etc.*) are already described in the literature, other applications are still unexplored; for example, taking advantage of the slow release mechanism provided by mesoporous silica structures.

Most of the properties and potential functionalities of these silica-polymer nanocomposites are dependent on a homogeneous distribution of the organic and inorganic components. For example, functionalized polymer nanoparticles can serve as a template to prepare silica hybrid films with a hierarchical structure to obtain superhydrophobic and amphiphobic coatings for self-cleaning surfaces. On the majority of described applications, the silica nanoparticles are incorporated in a polymer matrix. Due to mixability issues of the silica with most polymers, this requires the surface functionalization of the silica to achieve a good compatibilization of the two components and, thus, avoid the aggregation of the silica particles in the final material. By using hybrid nanoparticles with a silica core and a polymer shell, one cannot only obtain perfectly homogeneous particle distributions, but further control the spacing between silica particles in the final material, which might be important in many applications (for example, in photoactive composites).

## Figures and Tables

**Table 1.** Typical silane coupling agents used in the preparation of hybrid silica-polymer materials.

Abbreviation	Name	Chemical Structure	References
MPS	3-Methacryloxypropyl trimethoxysilane	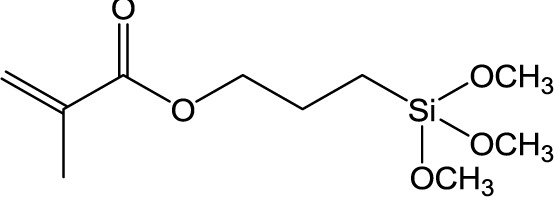	[9,13–18]
GPTMS	Glycidoxypropyltrimethoxysilane	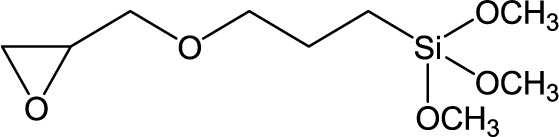	[4,19]
CPS	4-((3-(Trimethoxysilyl) propoxy)methyl)-1,3-dioxolan-2-one	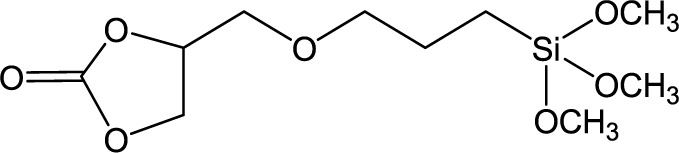	[20]
MPTES	3-Methacryloxypropyl triethoxysilane	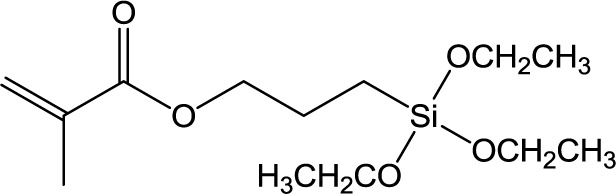	[21]
PATMS	*N*-[3-(trimethoxysilyl) propyl]aniline	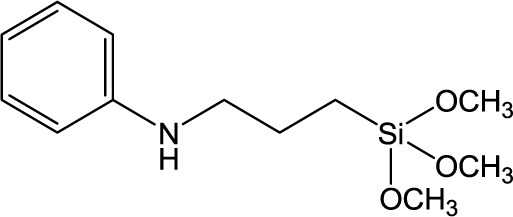	[22]
APTES	3-Aminopropyl triethyoxysilane	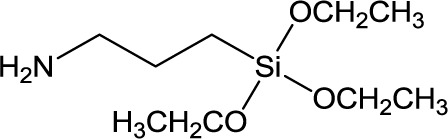	[6]

**Table 2.** Examples of methods for the preparation of hybrid polymer-silica materials with the respective organic composition.

Method of preparation	Organic composition	References
Blending	Poly(acrylate) (PA)	[12,23,24]
	Poly(methyl methacrylate) (PMMA)	[17]

Polymerization	Poly(methyl methacrylate-butyl acrylate) (P(MMA-BA))	[25–27]
	Poly(methyl acrylate) (PMA)	[14]
	Poly(butyl methacrylate) (PBMA)	[9,13]
	Poly(styrene-butyl acrylate) (P(S-BA))	[28]
	Poly(styrene) (PS)	[16]
	Poly(methyl methacrylate-butyl acrylate-methacrylic acid) (P(MMA-BA-MAA)	[29]

Sol-gel	Poly(styrene) (PS)	[30,31]
	Poly(styrene-butyl acrylate-acrylic acid) (P(S-BA-AA))	[32]
	Poly(methyl methacrylate) (PMMA)	[33]

## References

[1] Zou H., Wu S., Shen J. (2008). Polymer/Silica nanocomposites: Preparation, characterization, properties, and applications. Chem. Rev.

[2] Ray S.S., Okamoto M. (2003). Polymer/layered silicate nanocomposites: A review from preparation to processing. Prog. Polym. Sci.

[3] Stöber W., Fink A., Bohn E. (1968). Controlled growth of monodisperse silica spheres in the micron size range. J. Colloid Interface Sci.

[4] Shang X.-Y., Zhu Z.-K., Yin J., Ma X.-D. (2002). Compatibility of soluble polyimide/silica hybrids induced by a coupling agent. Chem. Mater.

[5] Zhang S., Yu A., Song X., Liu X. (2013). Synthesis and characterization of waterborne UV-curable polyurethane nanocomposites based on the macromonomer surface modification of colloidal silica. Prog. Org. Coat.

[6] Nazir T., Afzal A., Siddiqi H.M., Ahmad Z., Dumon M. (2010). Thermally and mechanically superior hybrid epoxy-silica polymer films via sol-gel method. Prog. Org. Coat.

[7] Yan W., Han Z.J., Phung B.T., Ostrikov K.K. (2012). Silica nanoparticles treated by cold atmospheric-pressure plasmas improve the dielectric performance of organic−inorganic nanocomposites. ACS Appl. Mater. Interfaces.

[8] Yan W., Han Z.J., Phung B.T., Faupel F., Ostrikov K.K. (2014). High-voltage insulation organic-inorganic nanocomposites by plasma polymerization. Materials.

[9] Ribeiro T., Baleizão C., Farinha J.P.S. (2009). Synthesis and characterization of perylenediimide labeled core-shell hybrid silica-polymer nanoparticles. J. Phys. Chem. C.

[10] Morales-Acosta M.D., Alvarado-Beltrán C.G., Quevedo-López M.A., Gnade B.E., Mendoza-Galván A., Ramírez-Bon R. (2013). Adjustable structural, optical and dielectric characteristics in sol-gel PMMA–SiO_2_ hybrid films. J. Non-Cryst. Solids.

[11] Rubio E., Almaral J., Ramírez-Bom R., Castaño V., Rodríguez V. (2005). Organic–inorganic hybrid coating (poly(methyl methacrylate)/monodisperse silica). Opt. Mater.

[12] Liao W., Teng H., Qu J., Masuda T. (2011). Fabrication of chemically bonded polyacrylate/silica hybrid films with high silicon contents by the sol-gel method. Prog. Org. Coat.

[13] Ribeiro T., Fedorov A., Baleizão C., Farinha J.P.S. (2013). Formation of hybrid films from perylenediimide-labeled core–shell silica–polymer nanoparticles. J. Colloid Interface Sci.

[14] Jethmalani J.M., Ford W.T. (1996). Diffraction of visible light by ordered monodisperse silica-poly(methyl acrylate) composite films. Chem. Mater.

[15] Li F., Zhou S., Gu G., You B., Wu L. (2005). Preparation and characterization of ultraviolet-curable nanocomposite coatings initiated by benzophenone/*n*-methyl diethanolamine. J. Appl. Polym. Sci.

[16] Xu D., Wang M., Ge X., Lam M.H.-W., Ge X. (2012). Fabrication of raspberry SiO_2_/polystyrene particles and superhydrophobic particulate film with high adhesive force. J. Mater. Chem.

[17] Chau J.L.H., Hsieh C.-C., Lin Y.-M., Li A.-K. (2008). Preparation of transparent silica–PMMA nanocomposite hard coatings. Prog. Org. Coat.

[18] Stojanovic D.B., Brajovic L., Orlovic A., Dramlic D., Radmilovic V., Uskokovic P.S., Aleksic R. (2013). Transparent PMMA/silica nanocomposites containing silica nanoparticles coating under supercritical conditions. Prog. Org. Coat.

[19] Isin D., Kayaman-Apohan N., Güngör A. (2009). Preparation and characterization of UV-curable epoxy/silica nanocomposite coatings. Prog. Org. Coat.

[20] Türünç O., Kayaman-Apohan N., Kahraman M.V., Menceloglu Y., Güngör A. (2008). Nonisocyanate based polyurethane/silica nanocomposites and their coating performance. J. Sol-Gel Sci. Technol.

[21] Watanabe M., Tamai T. (2006). Acrylic polymer/silica organic–inorganic hybrid emulsions for coating materials: Role of the silane coupling agent. J. Polym. Sci. A.

[22] Weng C.-J., Chen Y.-L., Jhuo Y.-S., Yi-Li L., Yeh J.-M. (2013). Advanced antistatic/anticorrosion coatings prepared from polystyrene composites incorporating dodecylbenzenesulfonic acid-doped SiO_2_@polyaniline core–shell microspheres. Polym. Int.

[23] Carneiro C., Vieira R., Mendes A.M., Magalhães F.D. (2013). Preparation and characterization of acrylic polymer nanocomposite films obtained from aqueous dispersions. J. Appl. Polym. Sci.

[24] Yang L., Zhou S., Gu G., Wu L. (2013). Film-forming behavior and mechanical properties of colloidal silica/polymer latex blends with high silica load. J. Appl. Polym. Sci.

[25] Dashtizadeh A., Abdouss M., Mahdavi H., Khorassani M. (2011). Acrylic coatings exhibiting improved hardness, solvent resistance and glossiness by using silica nano-composites. Appl. Surf. Sci.

[26] Fielding L.A., Tonnar J., Armes S.P. (2011). All-acrylic film-forming colloidal polymer/silica nanocomposite particles prepared by aqueous emulsion polymerization. Langmuir.

[27] Mizutani T., Arai K., Miyamoto M., Kimura Y. (2006). Application of silica-containing nano-composite emulsion to wall paint: A new environmentally safe paint of high performance. Prog. Org. Coat.

[28] Schmid A., Tonnar J., Armes S.P. (2008). A new highly efficient route to polymer-silica colloidal nanocomposite particles. Adv. Mater.

[29] Mizutani T., Arai K., Miyamoto M., Kimura Y. (2006). Preparation of spherical nanocomposites consisting of silica core and polyacrylate shell by emulsion polymerization. J. Appl. Polym. Sci.

[30] Niu L., Xia Z., Lei L., Zhang Y., Zhong L. (2013). Sol-gel process of alkoxysilane in emulsifier-involved aqueous emulsions: A one-pot synthetic route to emulsions of core-shell composite particles and their applications. J. Appl. Polym. Sci.

[31] Mammen L., Deng X., Untch M., Vijayshankar D., Papadopoulos P., Berger R., Riccardi E., Leroy F., Vollmer D. (2012). Effect of nanoroughness on highly hydrophobic and superhydrophobic coatings. Langmuir.

[32] Zhou X., Shao H., Liu H. (2013). Preparation and characterization of film-forming raspberry-like polymer/silica nanocomposites via soap-free emulsion polymerization and the sol-gel process. Colloid Polym. Sci.

[33] Palkovits R., Althues H., Rumplecker A., Tesche B., Dreier A., Holle U., Fink G., Cheng C.H., Shantz D.F., Kaskel S. (2005). Polymerization of W/O microemulsions for the preparation of transparent SiO_2_/PMMA nanocomposites. Langmuir.

[34] Xu X., Asher S.A. (2004). Synthesis and utilization of monodisperse hollow polymeric particles in photonic crystals. J. Am. Chem. Soc.

[35] Luna-Xavier J.-L., Guyot A., Bourgeat-Lami E. (2002). Synthesis and characterization of silica/poly (methyl methacrylate) nanocomposite latex particles through emulsion polymerization using a cationic Azo initiator. J. Colloid Interface Sci.

[36] Zhang K., Chen H., Chen X., Chen Z., Cui Z., Yang B. (2003). Monodisperse silica-polymer core-shell microspheres via surface grafting and emulsion polymerization. Macromol. Mater. Eng.

[37] Tiarks F., Landfester K., Antonietti M. (2001). Silica nanoparticles as surfactants and fillers for latexes made by miniemulsion polymerization. Langmuir.

[38] Zhou J., Zhang S., Qiao X., Li X., Wu L. (2006). Synthesis of SiO_2_/poly(styrene-co-butyl acrylate) nanocomposite microspheres via miniemulsion polymerization. J. Polym. Sci. Part A Polym. Chem.

[39] Perruchot C., Khan M.A., Kamitsi A., Armes S.P. (2001). Synthesis of well-defined, polymer-grafted silica particles by aqueous ATRP. Langmuir.

[40] Ohno K., Morinaga T., Koh K., Tsujii Y., Fukuda T. (2005). Synthesis of monodisperse silica particles coated with well-defined, high-density polymer brushes by surface-initiated atom transfer radical polymerization. Macromolecules.

[41] Schrade A., Landfester K., Ziener U. (2013). Pickering-type stabilized nanoparticles by heterophase polymerization. Chem. Soc. Rev.

[42] Zhang W.H., Fan X.D., Tian W., Fan W.W. (2012). Polystyrene/nano-SiO_2_ composite microspheres fabricated by Pickering emulsion polymerization: Preparation, mechanisms and thermal properties. eXPRESS Polym. Lett.

[43] Yin D., Zhang Q., Zhang H., Yin C. (2010). Fabrication of covalently-bonded polystyrene/SiO2 composites by Pickering emulsion polymerization. J. Polym. Res.

[44] Ma H., Luo M., Sanyal S., Rege K., Dai L.L. (2010). The one-step Pickering emulsion polymerization route for synthesizing organic-inorganic nanocomposite particles. Materials.

[45] Soleimani M., Haley J.C., Majonis D., Guerin G., Lau W., Winnik M.A. (2011). Smart polymer nanoparticles designed for environmentally compliant coatings. J. Am. Chem. Soc.

[46] Taylor J.W., Winnik M.A. (2004). Functional latex and thermoset latex films. J. Coat. Technol. Res.

[47] Peruzzo P.J., Anbinder P.S., Pardini O.R., Vega J., Costa C.A., Galembeck F., Amalvy J.I. (2011). Waterborne polyurethane/acrylate: Comparison of hybrid and blend systems. Prog. Org. Coat.

[48] Zhang S., Liu R., Jiang J., Yang C., Chen M., Liu X. (2011). Facile synthesis of waterborne UV-curable polyurethane/silica nanocomposites and morphology, physical properties of its nanostructured films. Prog. Org. Coat.

[49] Qian Z., Zhang Z., Song L., Liu H. (2009). A novel approach to raspberry-like particles for superhydrophobic materials. J. Mater. Chem.

[50] Liang J., Wang L., He L., Sun S. (2013). Pyridine-containing block copolymer/silica core-shell nanoparticles for one-step preparation of superhydrophobic surfaces. Phys. Chem. Chem. Phys.

[51] Xiong D., Liu G., Duncan E.J.S. (2013). Robust amphiphobic coatings from bi-functional silica particles on flat substrates. Polymer.

[52] Jiang C., Zhang Y., Wang Q., Wang T. (2013). Superhydrophobic polyurethane and silica nanoparticles coating with high transparency and fluorescence. J. Appl. Polym. Sci.

[53] Wang S.-D., Shu Y.-Y. (2013). Superhydrophobic antireflective coating with high transmittance. J. Coat. Technol. Res.

[54] Winnik M.A. (1997). Latex film formation. Curr. Opin. Colloid Interface Sci.

[55] Ye X., Farinha J.P.S., Oh J.K., Winnik M.A., Wu C. (2003). Polymer diffusion in PBMA latex films using a polymerizable benzophenone derivative as an energy transfer acceptor. Macromolecules.

[56] Wang Y., Winnik M.A. (1993). Polymer diffusion across interfaces in latex films. J. Phys. Chem.

[57] Feng J., Winnik M.A. (1997). Effect of water on polymer diffusion in latex films. Macromolecules.

[58] Winnik M.A., Wang Y.C., Haley F. (1992). Latex film formation at the molecular level: The effect of coalescing aids on polymer diffusion. J. Coat. Technol.

[59] Kawaguchi S., Odrobina E., Winnik M.A. (1995). Non-ionic surfactant effects on polymer diffusion in poly (butyl methacrylate) latex films. Macromol. Rapid Commun.

[60] Juhué D., Wang Y.C., Winnik M.A. (1993). Influence of a coalescing aid on polymer diffusion in poly(butyl methacrylate) latex films. Makromol. Chem. Rapid Commun.

[61] Farinha J.P.S., Martinho J.M.G., Kawaguchi S., Yekta A., Winnik M.A. (1996). Latex film formation probed by nonradiative energy transfer: Effect of grafted and free poly(ethylene oxide) on a poly(n-butyl methacrylate) latex. J. Phys. Chem.

[62] Kim H.B., Winnik M.A. (1994). Effect of surface acid group neutralization on interdiffusion rates in latex films. Macromolecules.

[63] Kim H.B., Winnik M.A. (1995). Factors affecting interdiffusion rates in films prepared from latex particles with a surface rich in acid groups and their salts. Macromolecules.

[64] Kobayashi M., Rharbi Y., Brauge L., Cao L., Winnik M.A. (2002). Effect of silica as fillers on polymer interdiffusion in poly(butyl methacrylate) latex films. Macromolecules.

[65] Wang Y.C., Zhao C.L., Winnik M.A. (1991). Specific carbon-carbon bond dissociation in highly excited alkanes: An example of mode selectivity in large molecules. J. Chem. Phys.

[66] Martinho J.M.G., Farinha J.P.S. (2013). Fluorescence decay methods for the characterization of latex film formation. Coat. Tech.

[67] Piçarra S., Afonso C.A.M., Kurteva V.B., Fedorov A., Martinho J.M.G., Farinha J.P.S. (2012). The influence of nanoparticle architecture on latex film formation and healing properties. J. Colloid Interface Sci.

[68] Oh J.K., Wu J., Winnik M.A., Craun G.P., Rademacher J., Farwaha R. (2002). Emulsion copolymerization of vinyl acetate and butyl acrylate in the presence of fluorescent dyes. J. Polym. Sci. A Polym. Chem.

[69] Kumar V., Misra N., Paul J., Bhardwaj Y.K., Goel N.K., Francis S., Sarma K.S.S., Varshney L. (2013). Organic/inorganic nanocomposite coating of bisphenol A diglycidyl ether diacrylate containing silica nanoparticles via electron beam curing process. Prog. Org. Coat.

[70] Chang C.-C., Oyang T.-Y., Hwang F.-H., Chen C.-C., Cheng L.-P. (2012). Preparation of polymer/silica hybrid hard coatings with enhanced hydrophobicity on plastic substrates. J. Non-Cryst. Solids.

[71] Zhou H., Wang H., Niu H., Gestos A., Lin T. (2013). Robust, self-healing superamphiphobic fabrics prepared by two-step coating of fluoro-containing polymer, fluoroalkyl silane, and modified silica nanoparticles. Adv. Funct. Mater.

[72] Raveh M., Liu L., Mandler D. (2013). Electrochemical co-deposition of conductive polymer–silica hybrid thin films. Phys. Chem. Chem. Phys.

[73] Moghal J., Reid S., Hagerty L., Gardener M., Wakefield G. (2013). Development of single layer nanoparticle anti-reflection coating for polymer substrates. Thin Solid Films.

[74] Yun J., Bae T.-S., Kwon J.-D., Lee S., Lee G.-H. (2012). Antireflective silica nanoparticle array directly deposited on flexible polymer substrates by chemical vapor deposition. Nanoscale.

[75] Ming W., Wu D., Benthem R.V., de With G. (2005). Superhydrophobic films from raspberry-like particles. Nano Lett.

[76] Schulz U. (2006). Review of modern techniques to generate antireflective properties on thermoplastic polymers. Appl. Opt.

